# Impact of Short Term Consumption of Diets High in Either Non-Starch Polysaccharides or Resistant Starch in Comparison with Moderate Weight Loss on Indices of Insulin Sensitivity in Subjects with Metabolic Syndrome

**DOI:** 10.3390/nu5062144

**Published:** 2013-06-10

**Authors:** Gerald E. Lobley, Grietje Holtrop, David M. Bremner, A. Graham Calder, Eric Milne, Alexandra M. Johnstone

**Affiliations:** 1 Obesity and Metabolic Health Division, Rowett Institute of Nutrition and Health, University of Aberdeen, Aberdeen, AB21 9SB, UK; E-Mails: d.bremner@abdn.ac.uk (D.M.B.); g.calder@abdn.ac.uk (A.G.C.); e.milne@abdn.ac.uk (E.M.); alex.johnstone@abdn.ac.uk (A.M.J.).; 2 Biomathematics and Statistics Scotland, Rowett Institute of Nutrition and Health, University of Aberdeen, Aberdeen, AB21 9SB, UK; E-Mail: Grietje@bioss.ac.uk

**Keywords:** insulin sensitivity, stable isotope kinetics, non-starch polysaccharides, resistant starch, weight loss, Minimal Models, metabolic syndrome

## Abstract

This study investigated if additional non-starch polysaccharide (NSP) or resistant starch (RS), above that currently recommended, leads to better improvement in insulin sensitivity (IS) than observed with modest weight loss (WL). Obese male volunteers (*n* = 14) were given an energy-maintenance (M) diet containing 27 g NSP and 5 g RS daily for one week. They then received, in a cross-over design, energy-maintenance intakes of either an NSP-enriched diet (42 g NSP, 2.5 g RS) or an RS-enriched diet (16 g NSP, 25 g RS), each for three weeks. Finally, a high protein (30% calories) WL diet was provided at 8 MJ/day for three weeks. During each dietary intervention, endogenous glucose production (EGP) and IS were assessed. Fasting glycaemia was unaltered by diet, but plasma insulin and C-peptide both decreased with the WL diet (*p* < 0.001), as did EGP (−11%, *p* = 0.006). Homeostatis model assessment of insulin resistance improved following both WL (*p* < 0.001) and RS (*p* < 0.05) diets. Peripheral tissue IS improved only with WL (57%–83%, *p* < 0.005). Inclusion of additional RS or NSP above amounts currently recommended resulted in little or no improvement in glycaemic control, whereas moderate WL (approximately 3 kg fat) improved IS.

## 1. Introduction

The increased prevalence of obesity in affluent countries has led to a rise in the associated morbidities of insulin resistance, with progression to type 2 diabetes, and increased cardiovascular risk [1,2]. This has increased pressure on primary health care costs [3] and resulted in a number of campaigns based on lifestyle changes to either reduce the level of obesity or at least achieve a scenario where the obesity has only limited impact on the co-morbidities [4,5,6]. The major lifestyle changes involve exercise and diet, especially those targeted at weight loss because even moderate reductions result in substantial improvements in insulin sensitivity (IS), maintenance of glycaemic control and lipaemia [7,8]. A wide range of approaches have been advocated to promote weight loss but there is good evidence that diets high in protein are particularly effective [9,10,11]. 

For many people, however, achieving or maintaining weight loss proves difficult and so dietary strategies to improve metabolic health independent of weight reduction have been proposed. Such strategies are commonly based on lowering either the rate or amount of glucose absorbed from a meal [12]. This may involve substitution of some of the easily digested carbohydrate with fermentable sources, often grouped under the term “fibre”, that are either partially or completely degraded in the large intestine to short chain fatty acids (SCFA). Absorbed SCFA may also have direct effects on mechanisms linked to IS, either by substitution of gluconeogenic demands or by enhancing glucose uptake by muscle and adipose tissue [13]. Soluble forms of fibre also slow the rate of glucose absorption from the small intestine and thus lower glycaemic excursion in the post-prandial state [14]. Structurally, fibre can be sub-divided broadly into two forms, resistant starch (RS) and non-starch polysaccharide (NSP). While both can be fermented in the colon, the exact site, rate of degradation and the nature of the SCFA released can vary [15] and these may affect IS and other metabolic parameters, either directly or indirectly. Indeed, a number of studies have reported that inclusion of fibre within the meal strategy [16] or use of either acute [17] or chronic [18] supplementation with RS does improve blood glucose control following a meal. It is less clear whether NSP is as effective as RS, however, and this is important because the former is the more prevalent form of fibre in Western diets. Furthermore, it is not known whether improvements in metabolic health associated with higher fibre intake at weight maintenance match those achieved with a modest reduction in body weight. These two questions were addressed in the current study. In view of the important contribution of amino acids as glucose precursors [19,20,21,22], a secondary aim was to assess the impact of the various dietary interventions on protein turnover and amino acid status. Additional questions linked to aspects of colonic health, as assessed by changes in the microbial populations [23] and enzyme activities [24] of stool samples were also addressed within the overall study but have been reported separately.

## 2. Experimental Section

This study was conducted according to the guidelines laid down in the Declaration of Helsinki and all procedures were approved by the North of Scotland Research Ethics Service (reference code 07/S0802/35, May 2007). Written consent was obtained from all participants. Subject information, nutrient intakes and fibre digestibility have been detailed elsewhere [23,24]. Briefly, 16 male volunteers were recruited, each with at least three symptoms of metabolic syndrome (including waist circumference > 102 cm, body mass index > 27 kg/m^2^ and fasting glucose > 6 mM). Two left the study for reasons unconnected with the protocol. The remainder (*n* = 14) completed 4 periods of dietary intervention, with all food supplied in cooked or ready-to-eat form. The volunteers were first provided for 7 day with a maintenance (M) diet of breakfast, lunch and dinner based on a 3-day rotating menu. The estimated metabolisable energy (ME) intake for this diet was based on 1.5× resting metabolic rate (measured in the overnight fasted state) for each individual and provided protein:carbohydrate:fat as 13:52:35 of calories. For the M diet, the RS and NSP intakes averaged 5.1 and 27.1 g/day [23]; the latter exceeds the current UK recommendations (18 g/day) but is similar to that proposed for men in other Northern European countries [25]. The second and third periods each lasted for 21 day and involved either the RS or the NSP diet, offered in a randomised order. With the additional NSP or RS given these two diets provided approximately double the recommended daily dietary fibre intake in the UK. Both diets provided the same ME intake and similar macronutrient composition as for the M period. The RS diet contained 60 g/day of Novelose 330 (type 3 resistant starch), such that average daily intake of RS was 25.4 g/day while the measured NSP intake was 16.0 g/day (but only 8 g/day from food tables used to devise the menus). The NSP diet contained additional bran and other structural carbohydrates and provided, on average, 41 g NSP per d while the RS intake was 2.5 g/day. Both the RS and NSP diets were provided on a 5-day rotation menu. The final period was a 21-day weight loss (WL) based on a high-protein strategy [26] with the total ME intake as 8.5 MJ/day for all volunteers and with 30:40:30 for the energy content as protein, carbohydrate and fat respectively, while NSP and RS were set at 25.1 and 2.9 g/day respectively. There was no washout period between any of the dietary periods and any food refusals were recorded daily. The ratio of soluble:insoluble NSP was calculated from composition tables to be approximately 1:6 for the NSP diet and 1:3 for the others, but subsequent chemical analysis of the diets yielded ratios from 1:3.8 to 1:7.6. At the end of each period body weight and fat content was measured by air-displacement plethysmography as described previously [11]. Stool samples were collected weekly and analyses of these for undigested fibre content and microbial populations have been reported elsewhere [23].

### 2.1. Kinetic Measurements

Endogenous glucose production (EGP) was quantified by [6,6-^2^H_2_]glucose infusions and this, in combination with infusion of [U-^13^C]glucose and measurement of plasma insulin, also allowed IS to be determined, based on adaptation of established Minimal Models. Protein turnover in the fasted state was estimated using [1-^13^C]leucine. All isotopes were obtained from Cambridge Isotope Laboratories (CK Gas Products, Hook, Hants, UK) and were of either metabolic grade or tested for sterility and pyrogenicity. On the last day of each dietary period the volunteers were fasted overnight (last meal before 2100 on the previous day) and attended the Human Nutrition Unit at 07:00. The volunteers voided before the following procedures. An 18 g Venflon cannula with a 3-way tap added was inserted in an antecubital vein and used for infusions. Another Venflon cannula (20 g) was inserted into a vein on the back of the contralateral hand in a retrograde direction and was used for “arterialized” blood sampling using a hot box set at 60 °C. With some volunteers it proved difficult to catheterise the hand vein and in these cases the sample catheter was inserted into an antecubital vein in the contralateral arm. Unless otherwise specified, all blood samples were taken into heparinised Monovette tubes (Sarstedt Ltd, Leicester, UK). A 9 mL sample was taken for overnight-fasted metabolic parameters and for determination of natural abundance for subsequent stable isotope procedures. Unless otherwise stated all subsequent blood samples were collected into heparinised tubes.

**Figure 1:**
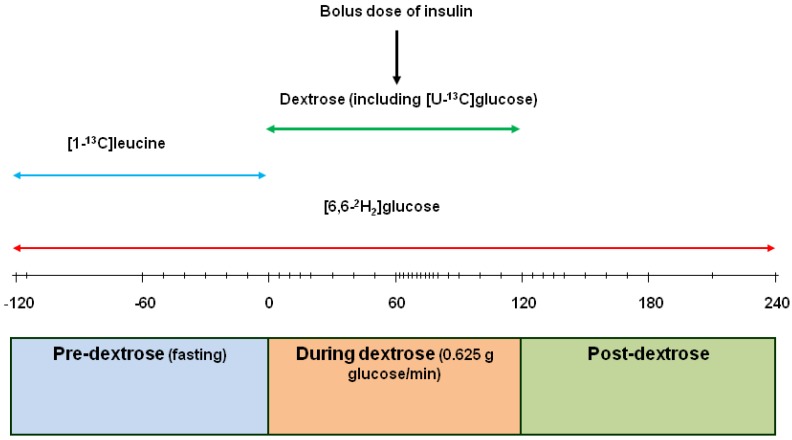
Time line (min) of the various infusions on the last day of each dietary period. The vertical dashes on the time line indicate sampling points. The [1-^13^C]leucine and [6,6-^2^H_2_]glucose were infused at tracer amounts, while the dextrose infusion represents a 75 g glucose challenge infused over 2 h. Insulin was injected at 0.04 IU/kg body weight (BW) at 60 min after start of dextrose infusion.

The protocol for the subsequent metabolic procedures is detailed in Figure 1. The design included a 2 h pre-measurement (−120 to 0 min) of endogenous glucose production and whole body protein breakdown under fasting conditions, then a 2 h measurement (0 to 120 min) of glucose rate of appearance (Ra) during a continuous intravenous infusion of glucose (37.5 g/h) followed by a 2h recovery period (120–240 min). Throughout the whole 6 h a 500 mL solution of [6,6-^2^H_2_]glucose (containing 160 µmol/kg BW; infusion rate 0.4 µmol/kg body weight (BW) per min) in sterile solution was infused (75 mL/h). At the start of the fasted measurements (time—120 min), the volunteers were given a weighed priming injection of [6,6-^2^H_2_]glucose (42 µmol/kg BW) plus [1-^13^C]leucine (4.2 µmol/kg BW) dissolved in 10 mL sterile saline and for the first 2h [1-^13^C]leucine (9.6 µmol/kg BW; infusion rate 0.08 µmol/kg BW per min) was included with the [6,6-^2^H_2_]glucose infusate. Arterialised blood samples (5 mL, plus 1 mL into EDTA) were taken at −60, −50, −40, −30, −20, −10 and −1 min with the subjects asked to place their hand in a hot box at 60 °C for the 5 min before each sample was taken. For the next 2 h (0 to 120 min) a 50% dextrose solution (2.78 M; Baxter Healthcare, Newbury, Berks, UK) containing [U-^13^C]glucose (6.45 mM; infusion rate 0.008 µmol/min) was infused (75 mL/h) through another port of the 3-way tap. Blood samples (2 mL, plus 1 mL into EDTA) were then taken at 5, 10, 15, 20, 30, 40, 50 59, 62, 64, 66, 68, 70, 75, 80, 90, 100, 110 and 120 min. At 60 min insulin (0.6 IU/mL, prepared by diluting 100 IU/mL Actapid with diluting medium, both from Novo Nordisk, Crawley, Sussex, UK) was injected at 0.04 IU/kg BW via the antecubital vein cannula and flushed through with 20 mL sterile saline. At 120 min the dextrose infusion was discontinued and blood samples (2 mL, plus 1 mL by syringe into EDTA) taken at 125, 130, 135, 140, 150, 160, 170, 180, 210, 240 min. At the end of the infusions the subjects emptied their bladder and the collected urine was weighed and 10 mL retained. Volunteers were allowed access to water during the infusion period. At the end of each infusion the pumps and tubing were calibrated gravimetrically and these values used to calculate actual rates of infusion.

On one separate occasion for each volunteer, after an overnight fast a bolus injection of [U-^13^C]glucose (12 µmol/kg) was given via a cannula inserted into an antecubital vein and blood samples (2 mL) taken from the same cannula at −5, 0.5, 1, 1.5, 2, 2.5, 3, 4, 5, 6, 7, 8, 9, 10, 12, 14, 16, 18, 20, 22, 24, 26, 28, 30, 35, 40, 45, 50, 55, 60, 70, 80, 90, 105, 120, 135, 150 min. The bolus injection was randomised between volunteers to encompass all the dietary treatments. 

### 2.2. Laboratory Analyses

All blood samples were immediately placed on ice and centrifuged at 1000× *g* for 15 min at 4 °C. Samples were either stored at −80 °C for later analyses or used fresh for immediate measurement of glucose concentration with a clinical analyser (Kone Ltd., Espoo, Finland) based on the hexokinase reaction (Thermo Fisher Ltd., Vantaa, Finland). Each sample measured in quadruplicate and care taken to prevent evaporation while the tubes were contained within the thermostatically-controlled well. For the bolus injection, glucose concentrations were determined on the samples taken at −5, 120, 135 and 150 min and the mean value used for the compartmental analysis.

All enrichments of [6,6-^2^H_2_] and [U-^13^C]glucose were determined as the penta-acetate derivatives and data expressed as tracer:tracee ratio (TTR), corrected for appropriate background. To ensure uniform peak areas, volumes of plasma (to the nearest 5 µL) were processed to yield 0.25 µmol glucose, based on the clinical analyser data. Plasma (20 to 70 µL) was added to 200 µL acetonitrile:ethanol mixture (2:1), vortex mixed for 30 s then centrifuged at 12,000× *g* for 5 min. The supernatant was dried at 90 °C under nitrogen. To the dry residue was added 75 µL acetic anhydride:pyridine (2:1) and the penta-acetate derivative formed by heating at 90 °C for 15 min, followed by drying and dissolution in 80 µL ethyl acetate. The [6,6-^2^H_2_]glucose was analysed on a Voyager mass spectrometer (Thermo Scientific, Hemel Hempstead, UK) coupled to a GC8000 Topgas chromatograph, with a 30 m × 0.25 mm × 0.25 µm Equity-1701 capillary column (Sigma-Aldrich, Gillingham, UK). The [6,6-^2^H_2_]glucose was measured under electron impact conditions with selective ion monitoring of fragment ions at *m/z* 200 and 202. The [U-^13^C]glucose (bolus injection) was determined under positive chemical ionization gas conditions on an Agilent 5973 Network MSD (Agilent Technologies, Manchester, UK) coupled to a 6890N gas chromatograph, with an Equity-1701 capillary column. Methane was used as the reagent gas and the ions at *m/z* 331 and 337 were monitored under selective ion monitoring conditions. 

Enrichments of [U-^13^C] glucose (from dextrose infusion) were determined by gas chromatograph combustion isotope ratio mass spectrometry (Delta plus XL fitted with a GC C II-III interface; Thermo Scientific, Winsford, Cheshire, UK). All penta-acetate derivatives were made to 400 µL with ethyl acetate and 1 µL injected in splitless mode onto a DB1701 column (30 m i.d., 0.322 film, 0.25 µM). All samples were measured in duplicate but if the standard deviation exceeded 0.001 atom % excess the analyses were repeated in duplicate. Background enrichments were based on the average of samples taken −20, −10 and 0 min before the start of the dextrose infusion (*i.e.*, during the [6,6-^2^H_2_]glucose plus [1-^13^C]leucine infusion). Urine was analysed where there were detectable amounts of glucose present (>0.2 mM). Enrichment of each infusate was also measured and averaged 99% (SE 0.7%) of those calculated from the weight of [U-^13^C]glucose added to the 50% dextrose solution.

The TTR for both leucine and its oxo-acid, 4-methyl-2-oxo-pentanoate (MOP), were determined from 0.9 mL plasma as previously described [27]. Amino acid concentrations were determined by isotope dilution with a gravimetric approach (to give µMolal) as described previously [28], but with two additions, homocysteine [29] and arginine, the latter was monitored at *m/z* 442 and 448 as a nitrile breakdown product of the derivative that eluted between lysine and histidine [30]. 

Plasma insulin and C-peptide concentrations were measured in duplicate by ELISA kits (Mercodia, Uppsala, Sweden). Insulin was measured on all samples for all diets. C-peptide was measured only for the M and WL treatments and was restricted to samples taken at 10 and 0 min before the start of the dextrose infusion and then at 10, 20, 30, 40, 50, 59, 75, 90, 100, 110, 120, 130, 140, 150, 160, 170, 180, 210, 240 min. Any duplicate samples with a CV greater than 10% were re-analysed. The coefficient of variation for intra- and inter- plate assays were 5.8% and 7.2% for insulin and 4.8% and 3.7% for C-peptide respectively. The factor 6.0 was used to convert insulin from mU/L to pM, where appropriate.

### 2.3. Calculations

The Homeostatic Model Assessment of insulin resistance (HOMA2-IR) [31] was calculated using a freely-available programme [32] based on the mean of the 7 fasted samples taken at 10 min intervals during the last hour of the [6,6-^2^H_2_]glucose plus [1-^13^C]leucine infusion. The sample taken 5 min after the bolus “prime” injection showed elevated glucose and insulin and was not used. Hepatic insulin sensitivity (HIS) [33] was calculated as 1000/(EGP × fasting plasma insulin).

Both incremental (AUCi) and total (AUCt) areas under the curve for glucose, insulin and C-peptide were calculated by the trapezoid method [34]. For comparisons between metabolites and hormones only identical samples were used, *i.e.*, for glucose and insulin all samples for all treatments were examined, while for insulin versus C-peptide the analysis was restricted to the sample times selected for C-peptide and for only M and WL diets.

The irreversible loss rates (ILR) of leucine, using plasma MOP as precursor, and EGP for glucose under fasting conditions were based on the 7 samples obtained during the hour preceding the dextrose infusion and for which a pseudo-plateau was reached. ILR (mmol/h) was calculated from

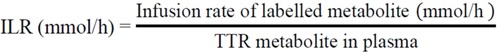



### 2.4. Insulin Sensitivity

#### 2.4.1. Rationale

For this study, a Minimal Model approach to measure IS was adopted. This has the advantage over the hyperinsulinaemic, euglycaemic clamp (HIEC) in that elements of pancreatic function and hepatic responsiveness could also be obtained. The IV rather than oral method was preferred because the former allows accurate definition of entry of glucose into the plasma, while the latter requires estimation of the rate of absorption of the glucose and necessitates another (and radioactive) tracer [35] in addition to the conventional ^13^C and ^2^H glucose tracers [36]. A model based on infusion rather than the conventional bolus injection [37] was developed to allow a greater amount of glucose to be supplied (75 g over the 2 h compared with the conventional intravenous bolus dose of 20 g) and that matches with the amount normally given as part of an oral glucose tolerance test for determination of impaired glucose tolerance or type 2 diabetes. Two models (Formal and Simple) were constructed to predict IS. 

**Figure 2:**
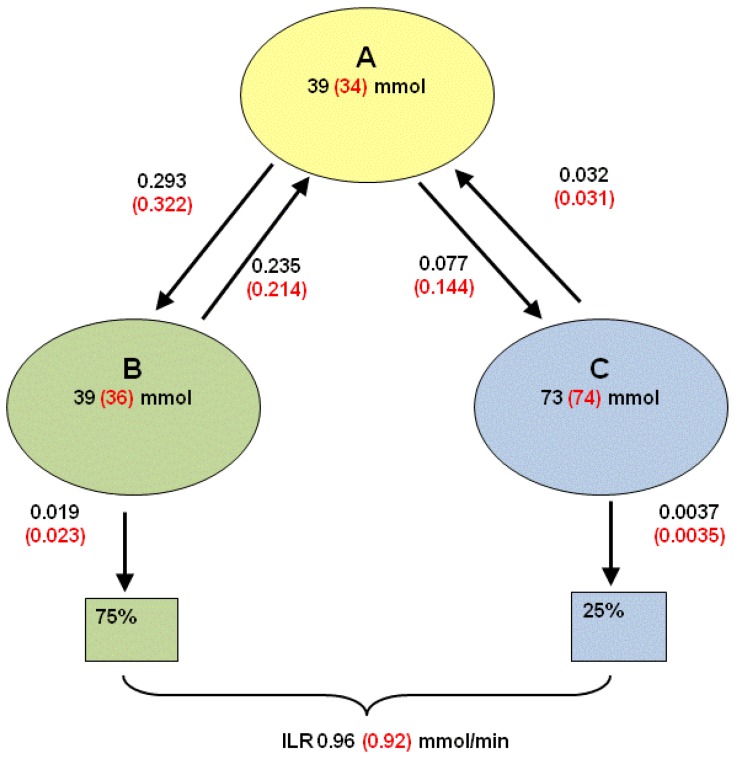
Three compartment model to describe transfers from a primary pool (**A**) to either a readily accessible (**B**) or less-readily accessible (**C**) pool. Values obtained from 14 volunteers with each measured on just one occasion in the overnight fasted state. Subjects were injected intravenously with a bolus dose of [U-^13^C]glucose and blood samples taken at intervals over 150 min. A three compartment model was fitted to the TTR of plasma glucose. Values in parentheses include all volunteers, other values exclude two volunteers for whom inadequate model fits were obtained. Values beside arrows are fractional rate constants (min^−1^).

Although the dextrose challenge was supplied at a constant rate, both the initial response to the infusion and the return to baseline conditions when the infusion was stopped are under non-steady state conditions. For this reason, estimates of the main parameters of the compartmental model shown in Figure 2 were needed. These parameters were then used for the fits to the equations derived for the Formal and Simple Models.

#### 2.4.2. Estimation of Compartmental Model Parameters from Bolus Dose [U-^13^C]glucose

The [U-^13^C]glucose enrichments were expressed as TTR. Due to imperfect mixing within the body, samples collected within 90 s of dosing were excluded. Fitting a 3-pool model to the enrichments gave the best fit (comparison of residual sum of squares using the *F*-test, *p* < 0.001):

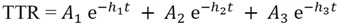

where *t* is the sampling time (min), and *A*_1_, *h*_1_, *A*_2_, *h*_2_, *A*_3_ and *h*_3_ are the parameters to be estimated. For 12 volunteers the accounted variance, adjusted for the degrees of freedom, exceeded 99.5%, while for the other two volunteers the accounted variance was 88.3% and 94.0%. The estimates of *A*_1_, *h*_1_, *A*_2_, *h*_2_, *A*_3_ and *h*_3_ were transformed into pool sizes and fractional rate constants by conventional procedures [38]. The notation used is as follows. *Q_x_* (mmol) refers to the size of pool *x*, and *k_x_* (min^−1^) denotes the fractional rate constant for this pool. Furthermore, *k_yx_* is defined as the fractional rate constant such that flows to pool *y* from pool *x* are given as *k_yx_* × *Q_x_*. The following assumptions [39] were made (see also Figure 2): EGP enters the system via the primary pool (pool A) that then exchanges glucose with pool B (rapid equilibration) and pool C (slow equilibration) but there is no exchange between these latter two pools. Finally, loss from the system occurs from pools B and C, where two scenarios were considered; the first assumes that 75% of the loss occurs from pool B and 25% from pool C [39], whereas the second assumes that 100% of the loss occurs from pool B.

#### 2.4.3. Adapting the 3-Pool Model Parameters to a 2-Pool Model

For estimation of EGP and ILR on experimental days a simpler model was used [34,40], based on 2 pools with EGP entering and ILR leaving the primary pool, and with the secondary pool acting as the peripheral compartment. To distinguish this model from the 3-pool model, the primary and secondary pools are referred to as pool 1 and pool 2, respectively. The volume (*V*, L) of the primary pool for each of the four experimental days was obtained from the amount of prime dose divided by the [6,6-^2^H_2_]glucose concentration in plasma measured 5 min after the dose was injected. For each volunteer the estimated volume was similar for each of the four diets and so the average was used. Finally, for each experimental day the pool sizes and rate constants of the 2-pool model were adjusted such that the ILR from the 2-pool model matched the ILR obtained from the continuous infusion of [6,6-^2^H_2_]glucose during baseline. In conjunction with *V* and the baseline glucose concentration, the pool size of the primary pool and *k*_01_ (min^−1^) were determined. Assuming *k*_2 _and *k*_01_/*k*_1_ are unchanged, then the remaining parameters were obtained. This approach resulted in parameters specific to each diet for each volunteer. On average, *k*_01_ = 0.0111/min (SEM 0.0003), *k*_21_ = 0.026/min (SEM 0.003), *k*_12_ = 0.034/min (SEM 0.002), *V* = 15.3 L (SEM 0.28; or 0.128 L/kg body weight), *Q*_1_ = 92.8 mmol (SEM 4.3), and *Q*_2_ = 74.7 mmol (SEM 8.3). 

#### 2.4.4. Concentrations of Various Glucose Species

For each experimental day, the measured total glucose concentration in plasma, denoted by *G*_tot_, is a composite of [6,6-^2^H_2_]glucose, [U-^13^C]glucose and natural abundance glucose, with the latter comprising glucose derived from endogenous sources plus that present in the dextrose infusate. Values for each glucose form were obtained from the enrichment (TTR) data. 

#### 2.4.5. Formal Model for Estimation of ILR and EGP during Dextrose Challenge

The two-compartmental model structures and assumptions detailed elsewhere [35,40,41] were taken as the starting point. Differences were that ILR was assumed to occur solely from the primary pool and, because several volunteers of this “at risk” population showed an increase in the *G_D2_* concentration during the first 30 to 60 min of the dextrose challenge, allowance was made for inhibition of fractional removal rate at high glucose concentrations. This was modelled as follows: let ILR denote the irreversible loss rate of all glucose species (mmol/min) combined, and assume that

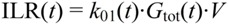

where *k*_01_(*t*) is modelled as

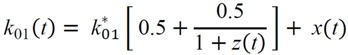
(1)
with 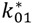 (min^–1^) the fractional removal rate constant at baseline. It is also assumed that

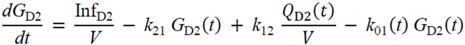
(2)

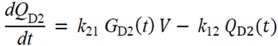
(3)
where *Q*_D2_ denotes the pool size (mmol) of the secondary pool for the [6,6-^2^H_2_]glucose and Inf_D2_ is the infusion rate (mmol/min) of the [6,6-^2^H_2_]glucose. Furthermore,

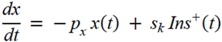
(4)

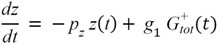
(5)
where 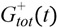 = *G_tot_*(*t*) − *G_tot_*(0), if *G_tot_*(*t*) > *G_tot_*(0); and 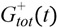 = 0, for *G_tot_*(*t*) ≤ *G_tot_*(0). *Ins^+^* were defined similarly. The use of *z*(*t*) in Equation (1) acts as inhibition on the fractional removal rate constant *k*_01_ under excess glucose load, where it is assumed that the maximum inhibition is limited to *k*_01_ being reduced by half. The rate of inhibition is modelled by means of parameter *g*_1_ (L/mmol/min) in Equation (5). The use of *x*(*t*) in equation (1) acts as enhancer of glucose removal due to high insulin concentrations, where the enhancement rate is modelled by means of *s_k_* (L/mU/min^2^) in Equation (4). This equation shows that for high insulin concentrations *x*(*t*) will be large and hence ILR will be enhanced. The parameters *p_x_* (min^−1^) and *p_z_* (min^−1^) model the gradual decay of the enhanced (or suppressed) removal with time.

*Q*_end_(*t*_0_) was set equal to *Q*_2_ from the adapted 2-pool model and *Q*_D2_(*t*_0_) was set equal to *Q*_end_(*t*_0_) *G*_D2_(*t*_0_)/*G*_end_(*t*_0_), where the start time *t*_0_ was taken to be 30 min prior to the start of the dextrose infusion. Furthermore, *x*(*t*_0_)=0 and *z*(*t*_0_)=0. Using R (R Foundation for Statistical Computing, Vienna, Austria, [42]), in conjunction with the gnlm (by Lindsey, J., [43]) and odesolve (CRAN repository on [42]) libraries, parameter estimates were obtained from minimising the weighted residual sum of squares, with weights given by the length of the interval between time points. The latter avoided the fit being dominated by samples taken close together. The percentage of variation accounted exceeded 80% for 48 of the 56 data sets. This was less for the remaining data, either due to variable *G*_D2_ concentrations throughout the time course of the study or due to the model failing to capture the rapid fluctuations observed in *G*_D2_ following the insulin bolus.

#### 2.4.6. Simple Model for Estimation of ILR and EGP during Dextrose Challenge

A simpler model, requiring fewer assumptions, has also been developed [35] but this involved use of a third (and radioactive) tracer which could not be adopted in the current study. Therefore the ILR and EGP calculations [35] were adapted for 2 tracers based on the observed changes in *G*_D2_ and *G*_end_. Again, the 2-pool model with ILR and EGP occurring from the primary pool was taken as starting point, where it was assumed that *k*_12_, *k*_21_ and *V* remain unchanged, but that ILR (and hence *k*_01_) and EGP vary with time. In steady state, ILR (covering losses of all glucose species) is given as ILR = *k*_01_ × *G*_tot_ × *V* and EGP will be equal to ILR. During the dextrose challenge, however, ILR is expected to increase and EGP to decrease. The ILR at time point *t* is modelled as

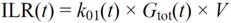
(6)
which allows for a variable fractional rate constant (for example, ILR may increase more rapidly than predicted from ILR(*t*) = 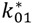
・ *G*_tot_(*t*)). The changes in the *G*_D2_ concentration and pool size are modelled as described in Equations (2) and (3).

For *δ* sufficiently small, *dQ*_D2_/*dt* can be written as [*Q*_D2_(*t* + *δ*) − *Q*_D2_(*t*))/*δ*], so that from Equation (3) *Q*_D2_ is obtained:

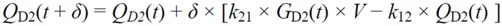

where *G_D2_*(*t*) is based on the observed [6,6-^2^H2]glucose concentration. Likewise, writing *dG_D2_*/*dt* = (*G_D2_*(*t* + δ) − *G_D2_*(*t*))/δ, *k_01_*(*t*) follows from equation (2):

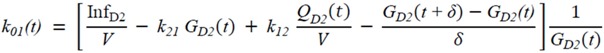



ILR(*t*) can now be calculated from Equation (6). To obtain EGP (*t*), the changes in the *G*_end_ concentrations are modelled as:

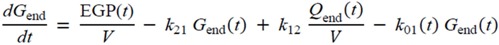
(7)

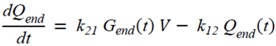
(8)


Analogous to *Q_D2_*, by writing *dQ*_end_/*dt* = (*Q*_end_(*t* + δ) − *Q*_end_(*t*))/*δ* and *dG*_end_/*dt* = (*G*_end_(*t* + *δ*) − *G*_end_(*t*))/*δ*, *Q*_end_ is obtained from Equation (8):

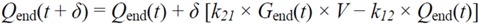

so that, from Equation (7):

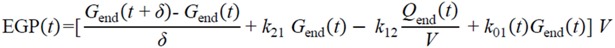



*Q*_end_(*t*_0_) was set equal to *Q*_2_ from the adapted 2-pool model and *Q*_D2_(*t*_0_) was set equal to *Q*_end_(*t*_0_) × *G*_D2_(*t*_0_)/*G*_end_(*t*_0_), where the start time *t*_0_ was taken to be 60 min prior to the start of the dextrose infusion. 

Implementation of the above was based on smoothed data (using the smooth.spline function in R on 20 degrees of freedom (df)) with concentrations estimated at every 0.01 min (so that *δ* = 0.01 min), so that estimates of ILR, EGP and *k*_01_ were obtained for every 0.01 min. Any negative outcomes were set equal to zero and all results were lightly smoothed (20 df). The detailed ILR, EGP and *k*_01_ were summarised by averaging over 60 min intervals.

Constraints by the local Ethics Committee on the amount of bolus insulin injected meant that only small perturbations in kinetic responses occurred and thus the action of insulin could not be separated from those of insulin plus glucose in the current experiment.

### 2.5. Statistical Analysis

Data are only considered for those volunteers (*n* = 14) who completed all four periods of the study. The design fixed the order of the maintenance and weight loss diets (periods 1 and 4 respectively) and only the order of the RS and NSP diets were randomised (across periods 2 and 3). For these two high-fibre diets a cross-check was performed that just compared these two diets with volunteer and period within volunteer as a random effect and period and diet, plus their interaction, as fixed effects. For all comparisons period was not significant so data from all four diets were analysed by Analysis of Variance (ANOVA), using Genstat 13th Edition Release 13.1 (VSN International Ltd., Hemel Hempstead, Herts, UK), with period effects ignored. For comparisons between diets, volunteer was treated as a random effect with diet as a fixed effect. For one volunteer, no [U-^13^C]glucose was included in the dextrose infusion during the M diet, and this was treated as a missing value where appropriate. 

The stability of the plateaux for the leucine kinetics and endogenous glucose production (EGP) under fasting conditions on each experimental day (*i.e.*, several sample times for each period for each volunteer) were tested by ANOVA with volunteer, and with period, sample time and their interaction nested within volunteer, as random effects and with diet and sample time, plus their interaction, as fixed effects.

Outcomes from the Simple model were aggregated over each of the four 60 min intervals for each volunteer period and analysed by ANOVA with volunteer, and with period, interval and their interaction nested within volunteer, as random effects, and with diet and interval, plus their interaction, as fixed effects. 

For all analyses, where significant main effects were observed (*p* < 0.05, all tests 2-sided) then difference between treatments were analysed by post-hoc *t*-test based on the relevant standard error of difference (SED), although only the SED for effect of diet are presented in the tables. Where reported in the text, the standard error of the mean (SEM) includes variation both within- and between-volunteers.

## 3. Results

### 3.1. Fasted Parameters (Table 1)

For the first 3 dietary interventions both mean body weight (121.1 kg) and body fat (49.0 kg) were similar, but declined by 6.1 kg and 4.9 kg (both *p* < 0.001) respectively following WL.

There was no effect of diet on plasma glucose concentration but, following the WL diet, insulin decreased by 35% (*p* < 0.001). In consequence, the HOMA2-IR also decreased with the WL diet (−27%, *p* < 0.001). A reduction in HOMA2-IR was also observed after the RS diet (−14%, *p* < 0.05 compared with the M and NSP diets). Similarly, β-cell function (HOMA2-%B) and IS (HOMA2-%S) both improved under WL compared with the other 3 diets. C-peptide was also 28% lower following WL (*p* < 0.001, compared with the M diet). The ratio of C-peptide:insulin, with both expressed as pM, increased between the M and WL diets (17.5 *vs.* 21.8, SED 1.76, *p* = 0.031).

**Table 1:** Effect of diet and time of sampling on various parameters ^1^ after an overnight fast following consumption of either a 7-day maintenance (M), or 21-day high non-starch polysaccharide (NSP), high resistant starch (RS) or weight loss (WL) diets by obese men (*n* = 14).

Parameter	M	NSP	RS	WL	SED	*P* ^4^ Diet	*P* ^5^ Time
*Glucose mM*	6.13	6.01	5.91	6.03	0.127	0.392	0.011
*Insulin pM*	94.9^ a^	95.0 ^a^	81.8 ^a^	59.4 ^b^	1.109	<0.001	0.214
*C-peptide pM*	1505	ND	ND	1085	83.1	<0.001	
*EGP μmol/min^ 2^*	1041 ^a^	995 ^a^	996 ^a^	923 ^b^	30.6	0.006	<0.001
*EGP* *μmol/min/kg FFM ^3^*	15.13 ^a^	14.56 ^a,b^	14.65 ^a^	13.72 ^b^	0.444	0.027	<0.001
*HOMA2-IR*	1.83 ^a^	1.83 ^a^	1.58 ^a^	1.16 ^b^	0.129	<0.001	
*HOMA2-%B*	93.2 ^a^	96.0^ a^	90.7^ a^	69.1 ^b^	5.15	<0.001	
*HOMA2-%S*	71.9^ a^	80.6^ a^	80.3^ a^	130.1^ b^	14.07	<0.001	
*HIS*	14.2 ^a^	15.9 ^a^	16.2 ^a^	28.3 ^b^	3.12	<0.001	

^1^ Mean values for glucose, and insulin based on 7 samples taken during 60 min prior to the dextrose infusion. C-peptide (only measured for M and WL diets and based on mean of samples taken at −10 and 0 min before start of dextrose infusion. ND, not determined). HOMA2, various Homeostatic model assessments (2) for IR, insulin resistance; %B, % pancreatic function; and %S, % insulin sensitivity [32]. HIS Hepatic Insulin Sensitivity (10^6^/(EGP × fasting plasma Insulin) [33]; ^2 ^EGP endogenous glucose production; ^3 ^FFM, fat-free mass; ^4^ For effect of diet based on ANOVA with volunteer as random effect, and with diet as fixed effects 39 residual degrees of freedom (df). SED values are presented for the effect of diet and differences between diets are represented by unlike superscripts within rows (*p* < 0.05); ^5^ For effect of time within sample day (for 7 samples taken between 60 and 120 min from start of infusion) by ANOVA, with random effects for volunteer and nested within volunteer, period, time and their interaction. Diet, time and their interaction were regarded as fixed effects; 78 residual degrees of freedom for testing the effect of time.

### 3.2. Basal Glucose and Leucine Metabolism

During the last hour of the fasted period, the plasma glucose concentration decreased by 2.2% (*p* < 0.001) across all diets. The altered glucose concentration, and thus pool size, may account partly for the slight increase (+8%; *p* <0.001) in [^2^H_2_]glucose TTR over the same time interval, although this may also involve return of label from intermediate pools [38]. EGP, expressed either in absolute units (*p* = 0.006) or corrected for fat free mass (FFM; *p* = 0.027), was lower with the 3 weeks of WL (Table 1) although, in the latter case, this was not significantly different from the NSP diet. For the WL diet the combination of lowered EGP and fasting insulin led to a higher estimated HIS (Table 1). 

During the last 60 min of [1-^13^C]leucine infusion the TTR increased for both Leu (+2.8%) and MOP (+2.2%) but the ratio of MOP:Leu enrichments was unaltered (data not shown). 

Diet had no effect on any of these responses. In terms of whole body kinetics (Table 2) diet also had no effect on ILR (equivalent to protein breakdown when intake is zero) based on plasma leucine enrichment (data not shown). The ILR (protein breakdown) based on plasma MOP did differ between diets, however, with lower values for NSP (*p* < 0.05) and WL (*p* < 0.001) compared with the M and RS diets (Table 2). These differences in ILR_MOP_ between treatments occurred despite similar overnight fasted plasma Leu concentrations and those of the other branch chain amino acids (Table 2). In contrast fasting plasma concentration of Trp was decreased (*p* < 0.01) on the WL diet as were Ala, Glu and Tyr, while Ser increased (*p* < 0.001). In addition, the concentration of Pro was lower (*p* < 0.01) with the RS compared with the NSP diet. 

**Table 2:** Overnight fasted irreversible loss rate (ILR; based on plasma MOP ^1^) and plasma amino acid concentrations (µMolal) at the end of each dietary intervention period ^2^ for 14 volunteers.

AA ^3^	Maint	NSP	RS	WL	SED	*P* ^4^ Diet
**ILRmop** μmol/min	200.6 ^a^	188.3 ^b^	198.0 ^a^	177.2 ^c^	4.91	<0.001
**Ala** μmol/kg	404.3 ^a^	382.5 ^a,b^	362.8 ^b^	299.1 ^c^	16.31	<0.001
**Arg** μmol/kg	101.9 ^a^	97.5 ^a,b^	94.7 ^b,c^	89.1 ^c^	3.53	0.007
**Asp** μmol/kg	4.0	3.7	3.4	3.0	0.40	0.076
**Cys** μmol/kg	300.1 ^a^	302.1 ^a^	287.9 ^a,b^	298.3 ^a^	3.63	0.002
**Gln** μmol/kg	587.4	593.5	578.8	566.3	13.70	0.233
**Glu** μmol/kg	98.0 ^a^	93.6 ^a^	90.1 ^a^	70.8 ^b^	5.73	<0.001
**Gly** μmol/kg	169.6	174.7	164.3	162.8	5.85	0.176
**Hcy** μmol/kg	6.7 ^a^	6.4 ^a,b^	6.1 ^b^	6.5 ^a^	0.19	0.017
**His** μmol/kg	79.5 ^a^	77.3 ^a,b^	75.4 ^b,c^	72.4 ^c^	1.64	<0.001
**Ile** μmol/kg	73.4	78.0	75.6	74.5	3.06	0.485
**Leu** μmol/kg	146.6	141.3	140.3	137.9	4.44	0.258
**Lys** μmol/kg	180.6	176.9	177.1	179.9	4.99	0.833
**Met** μmol/kg	29.1 ^a^	27.1 ^b^	25.9 ^b,c^	24.3 ^c^	0.87	<0.001
**Phe** μmol/kg	65.9 ^a^	61.1 ^b^	62.4 ^b^	59.5 ^b^	1.44	<0.001
**Pro** μmol/kg	229.3^a^	203.6^a^	179^b^	171.5^b^	7.83	<0.001
**Ser** μmol/kg	85.7 ^a^	85.1 ^a^	82.7 ^a^	94.1 ^b^	2.36	<0.001
**Thr** μmol/kg	117.4	114.6	117.2	119.5	5.19	0.829
**Trp** μmol/kg	58.6 ^a^	58.9 ^a^	59.9 ^a^	54.0 ^b^	1.50	0.001
**Tyr** μmol/kg	85.2 ^a^	78.7 ^b^	77.8 ^b^	72.0 ^c^	2.54	<0.001
**Val** μmol/kg	248.2	242.3	244.1	253.7	6.61	0.336

^1 ^MOP, 4-methyl-2-oxopentanoate (oxo-acid of leucine); ^2^ 7 day maintenance (M), or 21 day high non-starch polysaccharide (NSP), high resistant starch (RS) or weight loss (WL) diets; ^3^ Ala (alanine), Arg (arginine), Asp (aspartic acid), Cys (cysteine + cystine), Gln (glutamine), Glu (glutamic acid), Gly (glycine), Hcy (homocysteine), His (histidine), Ile (isoleucine), Leu (leucine), Lys (lysine), Met (methionine), Phe (phenylalanine), Pro (proline), Ser (serine), Thr (threonine), Trp (tryptophan) Tyr (tyrosine), Val (valine); ^4^* p*-value for Diet obtained from ANOVA, with subject as random effect and diet as fixed effect, 39 residual d.f. Data in rows with unlike superscripts differ by *p* < 0.05. Comparison of NSP v RS was also conducted by 2-way ANOVA with volunteer as random effect and diet and period, and their interaction, as fixed effects, 12 residual d.f. This is the only part of the design that was randomised cross-over and there were no significant effects of period (order).

### 3.3. Bolus [^13^C]Glucose Injections

Data for the 3-compartment model [39] fitted to the plasma enrichment curves following an intravenous injection of [^13^C]glucose for each volunteer in the overnight fasted state are shown in Figure 2. For two volunteers the 3-model fit gave small estimated primary pools, possibly due to incomplete mixing at the site of injection. For the other volunteers, the outflow from pool A to the rapid-equilibration pool (*k*_ba_) varied from 0.143 to 0.589 per min compared with 0.030 to 0.0.201 per min for the slow-equilibration pool (*k*_ca_). Such variation required that individual parameters were fitted for each subject. Overall the mean values for *k*_ca_ were similar to those observed for young, lean subjects (0.047/min) while *k*_ba_ was much lower than the 1.09/min reported previously [39]. The *k*_ba_ has been associated with insulin-independent transfers of glucose and the lower values may reflect inhibited uptake in the current obese subjects. The half-life of pool B ranged from 2 to 7 min (average 2.9 min) while that of pool C varied from 12 to 99 min (average 20 min). This means, on average, that >1 h would be needed to achieve a new equilibrium in the transition between steady states and that under non-steady state conditions, as in the current study, transfers from pools B and C would impact on measurements in pool A, which includes the sampled plasma.

The 3-compartment model parameters were simplified to those of a 2-compartment model by merging the first two pools and assuming that both EGP and ILR occur from the primary pool [35,40]. This formed the basis for subsequent estimation of EGP and ILR during the dextrose challenge. One outcome was that the model parameters for the two subjects that had a small estimated primary pool based on the 3-compartment model were now within the range observed for the other volunteers. For this reason the 2-compartment model was considered more robust. The fractional rate constants between pools 1 and 2 averaged *k*_21_ = 0.026 (SEM 0.003) per min and *k*_12_ = 0.034 (SEM 0.002) per min and these are lower than those reported for lean subjects (0.05 and 0.07 per min, respectively) [35].

### 3.4. Responses to Dextrose and Post-Dextrose Recovery

Mean concentrations for plasma glucose and insulin for each hour both during and following the dextrose infusion across diets are given in Table 3.

Although the amount of dextrose infused was identical for all treatments across all volunteers, there was a tendency (*p* < 0.10) for the WL diet to have higher glucose concentrations (5%–8%) 60 to 120 min into the dextrose infusion compared with the other three diets, with this effect more pronounced (*p* = 0.047) when expressed as changes in proportion to baseline glucose concentration. Over the same period, plasma insulin concentrations were lower (−37%, *p* < 0.001) for WL compared with the other 3 diets. Insulinaemia continued to remain lower (−41%, *p* < 0.001) during the first hour post-dextrose period for the WL diet and was still reduced, compared with the M diet, during the second hour post-dextrose (−50%, *p* = 0.021).

The greater C-peptide to insulin ratio for the WL versus M periods at fasting (Table 1) was maintained for the two diets both during and after the dextrose challenge (Table 3). When adjusted for the initial fasting value, however, the AUCi for C-peptide were not different between the WL and M diets for the 2 h either during or following the dextrose infusion (data not shown).

**Table 3:** Average ^1^ glucose (mM), insulin (pM) and C-peptide to insulin molar ratios for 60 min intervals for the 2 h during and 2 h after dextrose infusion in 14 volunteers after an overnight fast following the dietary interventions ^2^.

	Time (min)	M	NSP	RS	WL	SED	*P* ^3 ^Time	*P* ^3^ Diet	*P* ^3 ^T × D
*Glucose*	0–60	10.49	10.46	10.46	10.74	0.38	<0.001	0.450	0.209
	60–120	13.67	14.04	13.84	14.74				
	120–180	8.34	8.56	8.51	8.90				
	180–240	4.81	4.63	4.67	4.79				
*Insulin*	0–60	294 ^a^	287 ^a,b^	294 ^a^	199 ^b^	45.6	<0.001	<0.001	0.001
	60–120	689 ^a^	352 ^a^	634 ^a^	437 ^b^				
	120–180	572 ^a^	601 ^a^	544 ^a^	353 ^b^				
	180–240	214 ^a^	185 ^a,b^	161 ^a,b^	107 ^b^				
*Cpep:Ins*	0–60	8.61 ^a^			11.37 ^b^	2.596	0.028	<0.001	0.017
	60–120	5.99			8.66				
	120–180	8.94			12.59				
	180-240	15.70 ^a^			26.26 ^b^				

^1^ Based on area under the curve (expressed per min) for each 60-min interval; ^2^ 7 day maintenance (M), or 21 day high non-starch polysaccharide (NSP), high resistant starch (RS) or weight loss (WL) diets; ^3 ^*p*-values from ANOVA with volunteer and nested within volunteer, period, time and their interaction as random effects, and with diet, time and their interaction as fixed effects; when main effects of diet or the time × diet interaction was significant post-hoc comparisons for diet were performed; SED values refer to main effects for diet; Values within rows with unlike superscripts are different at *p* < 0.05.

### 3.5. Endogenous Glucose Production and Glucose ILR

**Figure 3:**
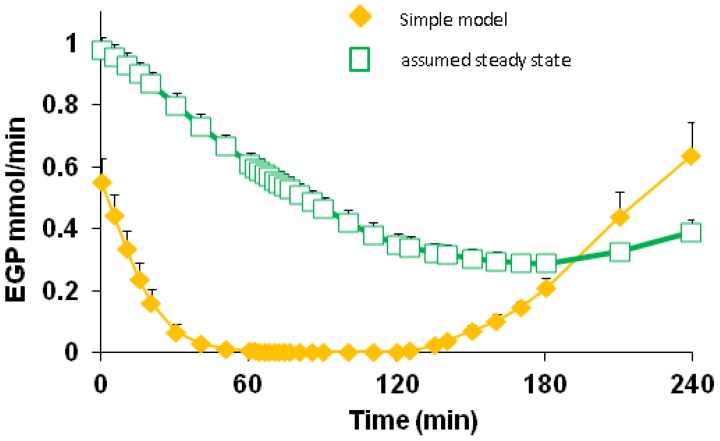
Comparison of calculated endogenous glucose production (EGP) based on either the Simple model, that allows for return of label to primary pool, or assuming steady state, where no allowance for label return is included. Data from the M diet, but the other diets gave similar patterns (not shown). The difference in calculated EGP at time 0 is because the Simple model allowed for the slow decline in plasma glycaemia during the 2 h fasting prior to the dextrose challenge. Differences in the decline and recovery of EGP over time again reflect that the Simple model allows for the return of label to the plasma pool while the steady state approach does not.

Based on the Simple model, estimated EGP approximately halved within 5 min of the start of dextrose infusion (data not shown) and was reduced to less than 10% (0.09 SEM 0.017) mmol/min) at 30 min into the dextrose infusion and remained low for the remaining 90 min of challenge (Table 4). The decline in EGP was more rapid than the change in the TTR of plasma [6,6-^2^H_2_]glucose (Figure 3) because the latter included label return from the slow-equilibration pool. Nonetheless, diet did not affect the rate of EGP suppression (*p* = 0.60).

**Table 4:** Kinetic parameters showing effect of diet on glucose dynamics and insulin sensitivity, as assessed by the Simple model, for 60 min intervals for the 2 h during and 2 h after dextrose infusion in 14 volunteers after an overnight fast following the dietary interventions ^1^.

Variable^2^	Time (min)	M	NSP	RS	WL	SED	*P* ^3 ^Time	*P* ^3 ^Diet	*P* ^3^ T × D
*EGP*	0–60	0.149	0.146	0.169	0.173	0.036	<0.001	0.601	0.966
	60–120	0.002	0.006	0.013	0.015				
	120–180	0.076	0.083	0.101	0.128				
	180–240	0.428	0.373	0.389	0.418				
*ILR*	0–60	1.95 ^a^	1.85 ^a,b^	1.94 ^a^	1.64 ^b^	0.11	<0.001	0.830	0.012
	60–120	3.74 ^a^	3.74 ^a^	3.73 ^a^	3.72 ^a^				
	120–180	3.07 ^a^	3.23 ^a,b^	3.20 ^a^	3.42 ^b^				
	180–240	1.40 ^a^	1.38 ^a^	1.38 ^a^	1.54 ^a^				
k*_01_*	0–60	0.0124^ a^	0.0117^ a^	0.0122^ a^	0.0101^ b^	0.0011	<0.001	0.929	0.047
	60–120	0.0184^ a^	0.0182^ a^	0.0182^ a^	0.0172^ a^				
	120–180	0.0261^ a^	0.0277^ a^	0.0268^ a^	0.0278^ a^				
	180–240	0.0194^ a^	0.0203^ a^	0.0207^ a^	0.0219^ b^				
*ILR/Ins*	0–60	8.83 ^a^	9.33^ a^	8.67^ a^	13.50^ a^	2.834	<0.001	0.003	<0.001
	60–120	0.045^ a^	0.049^ a^	0.046^ a^	0.075^ a^				
	120–180	9.17^ a^	9.34^ a^	9.84^ a^	17.84^ b^				
	180–240	11.84^ a^	13.50^ a^	13.00^ a^	27.84^ b^				

^1^ Seven day maintenance (M), or 21 day high non-starch polysaccharide (NSP), high resistant starch (RS) or weight loss (WL) diets; ^2 ^EGP, endogenous glucose production (mmol/min); ILR, irreversible loss rate of glucose (mmol/min^-1^); *k*_01_, fractional rate of disappearance of glucose (/min); ILR/Ins, irreversible loss rate per unit insulin (μmol/L/pmol per min); ^3 ^*p*-values from ANOVA with volunteer and nested within volunteer, period, time and their interaction as random effects, and with diet, time and their interaction as fixed effects. When diet or the time by diet (T × D) interaction was significant post-hoc comparisons for diet were performed. SED values refer to main effects for diet. Values within rows with unlike superscripts are different at *p* < 0.05.

At the same times (5 and 30 min of dextrose infusion), observed glucose concentrations increased by 22% and 86%, respectively, while plasma insulin was raised by 93% and 347% with C-peptide still 74% higher than baseline at 30 min. The rapidity of these changes, combined with the time intervals selected between samples, did not allow adequate resolution of the separate contributions of glucose and insulin to EGP suppression. The high plasma glucose and(or) insulin concentrations in the period after cessation of the dextrose infusion for some volunteers (Figure 3) continued to suppress EGP, as shown by the slow rate of decrease in the plasma [^13^C]glucose TTR. Indeed, 1.5 h after the dextrose infusion ceased, EGP was, on average, 410 (SEM 70) μmol/min, <50% of the basal rate. By this time plasma glucose concentrations were lower (−24%) than fasting glycaemia, while plasma insulin and C-peptide both remained elevated (+93% and +65%, respectively) compared with pre-dextrose infusion values.

Based on the Simple model, the ILR of glucose (Table 4) was elevated markedly during 60–120 min of dextrose infusion (3.73 mmol/min, SEM 0.19), dominated by exogenous glucose supply (3.47 mmol/min), and remained elevated during the first hour post-dextrose. The WL diet gave a lower ILR 0–60 min into the dextrose infusion, compared with the M (*p* = 0.004) and RS (*p* = 0.006) diets. This pattern was reversed for the first hour post-dextrose, with ILR remaining elevated on the WL diet compared with M and RS (*p* = 0.001 and *p* = 0.043 respectively). A similar pattern was observed for the fractional rate of removal, *k*_01_, which was lower for WL during the first h of dextrose infusion but remained higher for the last h of the post-glucose period.

### 3.6. Insulin Sensitivity

IS, an index of the responsiveness of peripheral tissue glucose disposal to the hormone, as determined by either the Formal or Simple models for the period during and post the dextrose infusion, improved during the period of weight loss compared with the other diets. For the Simple model, IS, assessed by glucose ILR divided by insulin concentration, improved by approximately two-fold on WL compared with the other three diets (*p* < 0.003; Table 4). This enhanced response resulted from a combination of increased ILR (Table 4) and lowered insulin (Table 3) with the WL diet. Similarly, with the Formal model, IS assessed as the response of glucose removal to the presence of insulin, s_k_, was increased for WL by 47%, 36% and 33% compared with M (*p* < 0.001), NSP and RS treatments (both *p* = 0.01), respectively (Table 5). Furthermore, IS remained elevated for the WL diet during the 2 h after the dextrose infusion ceased (data not shown). For neither model was there any alteration of IS for the RS or NSP diets in comparison with the M diet (Table 4, Table 5). In addition, with the Formal model approach no parameter, other than s_k_, was altered by diet (Table 5). 

**Table 5:** Estimated parameters ^1^ for the Formal model following the dietary interventions ^2^.

Parameter	M	NSP	RS	WL	SED	*P* ^3^ Diet
*p_x_* (/min)	16.71	12.40	15.13	10.61	3.38	0.286
*s_k_* (L/mU/min^2^)	7.43 ^a^	8.06 ^a^	8.20 ^a^	10.93 ^b^	1.17	0.024
*p_z_* (/min)	1.30	29.80	32.45	1.56	23.53	0.867
*g_1_* (L/mmol//min)	153	122	157	225	54.2	0.240

^1^* p_x_*, reflects gradual dimunition of insulin effect with time; *s_k_*, enhanced glucose removal due to insulin; *p_z_*, reflects gradual dimunition of glucose effect with time; *g_1_*, reduced fractional glucose removal due to high glucose concentration; ^2^ Seven day maintenance (M), or 21 day high non-starch polysaccharide (NSP), high resistant starch (RS) or weight loss (WL) diets; ^3^ Based on ANOVA with volunteer as random effect and diet as fixed effect, 39 df. Post hoc *t*-test was performed when diet was significant (*p* < 0.05). SED values refer to main effect for diet. Values within rows with unlike superscripts are different at *p* < 0.05.

Both models indicated there was an upper limit for glucose removal, particularly in the early phase of dextrose infusion when changes in glucose concentration exceed the response in insulin. Although the two models have different inherent advantages and disadvantages, both gave similar ILR and fractional removal rates, and showed the same effect of diet on the outcomes of interest (Table 4, Table 5).

## 4. Discussion

The challenge to health that accompanies obesity has led to a plethora of recommended approaches to reduce risks, including those associated with impaired IS. Among the proposed solutions is increased dietary fibre intake [44] or strategies that reduce body fat [8]. The current study addressed whether consumption of additional fibre, in the form of either NSP or RS, above that currently recommended for colonic health would improve metabolic health and IS in obese subjects who were weight-stable and how this would compare with improvements produced by a mild weight loss. An intervention of only three weeks was sufficient to increase IS following weight loss while improvements with RS or NSP, over a similar period, were marginal or non-existent.

### 4.1. Protein Dynamics and Aminoacidaemia

WL is well-known to improve metabolic health and IS [6,7,8] and so provides a good reference point for other dietary manipulations. In the current study a high-protein diet was used to support WL as this has proved effective in several laboratories and also ensures that lean mass is maintained [9,10,11]. This is important because amino acids provide an important precursor source for gluconeogenesis, particularly in the fasted state [22]. Indeed, when protein catabolism is increased so is the contribution of gluconeogenesis to glucose production and this is responsive to altered IS [20]. In practice, the RS diet had no effect on protein breakdown while both the NSP and WL diet caused a small decrease in the fasted condition. Whether this might also lead to reduced gluconeogenesis was not tested, although total EGP expressed per unit lean mass did decline. Alanine and glutamine are considered the most important non-essential amino acids that act as glucose precursors in humans [45] but only plasma concentrations of the former decreased after 3 weeks on the high protein WL diet. Interestingly, alanine also decreased with the RS diet, albeit to a lesser extent. Nonetheless, these indirect observations would suggest that the contribution of amino acids to glucose synthesis in the fasted state is probably similar between the various diets examined in the current study. 

### 4.2. Endogenous Glucose Production

Insulin regulates glycaemia by stimulation of glucose uptake by peripheral tissues and reduction of endogenous glucose production (EGP) by the liver. Either or both processes can be inhibited during IS. Hyper-insulinaemia in the portal vein suppresses both glycogenolysis and gluconeogenesis, the two major contributors to EGP, in non-diabetic subjects [46,47,48]. Elevated plasma glucose can also reduce EGP, mainly by suppression of glycogenolysis [35]. In the overnight fasted state, where neither insulin nor glucose are elevated, neither additional RS nor NSP impacted on EGP, which supports other observations [49,50]. In contrast, for the fasted state, the WL regime resulted in lowered EGP, although the 11% reduction was less than reported elsewhere [51]. Several factors may contribute to this decreased EGP. For example, the observed reduction in whole body protein breakdown may limit the amount of amino acid liberated from muscle and that contribute approximately 50% towards hepatic gluconeogenesis [52]. Alternatively, during WL mobilisation of body fat, production of ketone bodies may provide alternative sources of energy [53] and reduce the need for glucose. 

### 4.3. C-Peptide and Insulin

High fibre intakes appear to exert little effect on overnight fasted plasma insulin [18,54], as also observed in the current study. Post-prandial responses in insulin and C-peptide secretion to altered dietary fibre intake have been varied, however. For example, no change was observed for the insulin AUC (both total and incremental) between a normal and high fibre diet (11 *vs.* 26.7 g/MCal), each supplied for 4 weeks [54]. In contrast, a 24 h prior supplementation with 60 g RS2 (as Novelose 260) reduced post-prandial excursions of both glucose and insulin [17], although C-peptide was unaltered. The same group reported that a 4 weeks supplementation with 30 g/day additional RS2 (as Hi-maize 260) also reduced post-prandial insulin AUC [18]. There was also an increase in the C-peptide:insulin ratio, which supported earlier evidence that more insulin is removed by the liver with viscous fibre diets [55]. In contrast, the current study did not find an effect of the RS diet, when provided for 3 weeks as Novelose 330 (RS3), in response to infused dextrose. These differences between studies are difficult to reconcile, although both the form in which RS was supplied and the response conditions employed (test meal of mixed nutrients *vs.* intra-venous infused dextrose) may be critical. Furthermore, there were also no differences in the AUC for insulin for the NSP diet, which supports an earlier report with high fibre diets [54].

Although neither form of dietary fibre altered insulin responses, there were marked reductions in plasma insulin concentrations with the high protein WL diet, under both basal and glucose-stimulated conditions. Protein is a potent insulin secretagogue and acts synergistically when supplied with glucose [56]. Nonetheless, even after 5-week on a high protein, weight maintenance diet subjects with type 2 diabetes had similar overnight fasted plasma insulin concentrations compared with normal protein intake [4]. The lowered fasting insulin (and C-peptide) observed in the current study, therefore, may be due to either the hypo-caloric state and (or) the effect of weight loss. The latter may be more critical because in a number of studies where subjects lost weight and were then weight-stabilised for 1–2 weeks there were substantial declines in fasting insulin concentrations [8,57,58], although this has not always been observed [7]. Differences in fasting insulin and C-peptide between diets confound interpretation of pancreatic and hepatic responses to the glucose challenge. For example although there was lowered insulin AUC_t_ and raised C-peptide:insulin ratio for WL compared with M, the incremental AUC for C-peptide was similar between the two diets. This may indicate that the response of the pancreas to the same glucose challenge was similar between the diets, even though the basal (fasting) parameters differed. Resolution of this issue requires use of more sophisticated models that define pancreatic responses under non-steady state conditions [40] and that allow for the different half-lives of C-peptide and insulin [59,60]. 

### 4.4. Diet and Insulin Sensitivity

Based on both the Formal and Simple models, peripheral IS altered in response to WL but not for either the RS or NSP diet. The improvement with WL ranged from 56% to 83%, dependent on the model selected, and was driven by lowered insulin concentrations rather than changes in either glycaemia or glucose disposal. These findings support a number of previous reports where responses to meals or glucose challenges have been investigated after weight loss [57,61] and with either maintained or improved glycaemia achieved at lowered insulin concentrations. Similarly, under conditions of hyper-insulinaemia the rate of glucose disposal is enhanced by weight loss in obese subjects [8,51,62]. Such improvements in glycaemic control by insulin can result from either increased disposal by peripheral (muscle) tissue [8,51] and (or) enhanced hepatic metabolism, whereby EGP is further suppressed while glucose uptake is maintained [51]. In the current study, suppression of EGP during WL exceeded 90% but this would only account for a decrease in hepatic output of approximately 1 mmol/min, insufficient to compensate for the rate of dextrose infusion (3.5 mmol/min) and, instead, increased glucose disposal occurred.

Current UK recommendations for healthy eating include increased fibre intake by inclusion of fruit, vegetables and whole grains in the diet. Changes in dietary pattern may also affect other factors that influence IS, including altered diet glycaemic index (GI) [63], substitution of digestible carbohydrate by fermentable fibre [64] and inclusion of soluble fibre that lowers glucose absorption [14]. These various factors have complicated resolution of the direct effects of fibre components on metabolic health [65]. In practice, the amount of additional RS or NSP added to the current diets represented only 7%–13% (w/w) of the total carbohydrate present with only limited impact on diet GI. Care was also taken to maintain a fixed proportion of calculated soluble:insoluble fibre for each menu day although, in practice, this was not fully achieved. Both the M and WL diets were considered to meet fibre needs for optimal colonic health [66] and the additional NSP or RS tested if improvements in metabolic health could be achieved through increased intake of either fibre source. This is particularly relevant in the case of RS as intake of this form of fibre is relatively low (5–10 g/day) in western diets and one form of supplemental RS2 has been shown to improve IS [18,67]. The lack of response in glucose metabolism to additional NSP agrees with earlier work where the dietary fibre was increased from 11 to 27 g/Mcal intake [53], a greater differential than used in the current study (10.4, 13.5, 13.0 and 14.5 g/Mcal for M, NSP, RS and WL diets respectively). The carefully controlled earlier study [54] used substitution with whole grain cereals, whole wheat bread, fruit and nuts to increase the fibre intake but found no change fasting glucose or insulin, nor differences in AUCi for either glucose or insulin. In contrast, increased cereal fibre intake (from 14 g/day as control to either 26 or 43 g/day) for 6 weeks did improve IS [50]. Interestingly, the improvement was similar for the two supplements and these increased total fibre intake from a control value of approximately 20 g/day to either 32 or 50 g/day [50], calculated equivalent to 10, 13.5, 16 g/Mcal (from [50]). This compares with the current study where total fibre ingestion with the M diet was 32 g/day, raised to 42 and 44 g/day with the RS and NSP supplements respectively [23]. The reported findings [50,54], in combination with lack of change in IS in the current study, would suggest that increasing NSP above current recommendations for northern European countries [25] will not improve metabolic health, although there may be benefits on IS for those with habitual low fibre intake [50].

The situation is less clear in the case of RS. When soluble fibre was increased from 15 to 50 g/day by inclusion of additional fruit and vegetables in the diets of subjects with type 1 diabetes for a 4-week period there were significant reductions in mean daily blood glucose concentrations and glycosylated haemoglobin (HbA_1c_) [16]. These diet changes lowered the GI from 90% to 70% and this, rather than the inclusion of soluble fibre, may have been the main cause of the improvement. Nonetheless, other reports have suggested that either acute (24 h) [17] or chronic (4 week) [18] supplementation with 40 g/day RS in the form of high amylose maize (HAM-RS2) led to improvements in aspects of glycaemic control in healthy volunteers. The chronic study [18] showed an improved IS of 14%, assessed by the HIEC clamp procedure, but no change in HOMA. This was supported by later studies from the same group on subjects with metabolic syndrome and where IS improved by 19% [67], although this interpretation is slightly confounded by differences at baseline for the placebo and RS groups. In contrast, in further studies neither acute ingestion (24 g HAM-RS per meal) in healthy subjects [68] nor 4-week ingestion (40 g/day) in overweight subjects [69] showed improvement in either oral IS (by minimal model analysis) or HOMA parameters, Interestingly, lower doses of HAM-RS2 (15 and 30 g/day, each for 4 week) has recently been reported to increase IS (assessed by an insulin-modified IVGTT) in overweight and obese males but not their female counterparts [70]. 

The above comparisons clearly demonstrate that even with the same (or similar) RS product a number of factors may influence whether IS can be improved [65]. The current study used retrograde RS-3 starch (manufactured by a cooking and cooling process) and was designed to test whether the increased fermentation to short chain fatty acids (especially propionate) in the caecum would enhance IS [17,65,71]. This form of RS was almost completely fermented (>96%) in all but two of the volunteers, with corresponding increases in the proportions of Eubacterium rectale, Ruminococcus bromii and Oscillibacter within the faecal bacteria [23]. Most of these changes were rapid, occurring within 3–4 days of the diet switch, and bacterial metabolite changes would be expected to occur over a similar period so that any direct effects exerted should be apparent after the 3 week on each diet. Despite this, the RS diet resulted in only a slight improvement in HOMA but with no change in IS as assessed by the IVGTT procedure adopted. Why differences in actions on IS exist between (or even within) forms of RS is not resolved. In some studies, the RS is provided as a supplement [18,69,70] and this may provoke changes in habitual intake (but insufficient to affect body weight). In contrast, the current volunteers were carefully managed with all food provided as prepared meals. As with any “live-out” study, however, it is impossible to eliminate consumption of additional foods and drinks. Detailed bacterial changes associated with ingestion of RS2 (in the form of HAM) by humans have not been characterised and these may differ between RS2 and RS3 sources. Health benefits of RS consumption, other than changes related to IS, have been reported for the gastrointestinal tract, including crypt mitotic location, gene expression and DNA methylation [72]. 

A further explanation for the apparent inconsistency in response to RS may relate to the methods used to determine IS. The initial studies with HAM-RS2 [18] adopted the HIEC approach and reported improvements of 14%. Use of Minimal Models, either in conventional form or as modified here, either found no response [69] or numerical improvements (RS +17% and NSP +15%) that did not reach statistical significance (current study). The intra-venous based Minimal Models involve insulin only released from the pancreas whereas the HIEC procedure involves a fixed exogenous supply plus pancreatic secretion. The additional biological variance associated with Minimal Models, although more closely simulating the natural state, may increase the difficulty in detecting small differences. Interestingly, the recently study with HAM-RS2 and use of an insulin-stimulated Minimal Model did observe significant responses but here the improvement in IS approached 50%. Nonetheless, within the current study any improvement with either NSP or RS3 was far less than the 37%–53% achieved by a modest 3-week weight loss.

## 5. Conclusions

The failure of advice from the medical profession, scientists and governments for people to lose weight has forced consideration of whether people can be fat but healthy (metabolically healthy but obese, MHO). Although a proportion of the population appears to be naturally MHO [73], which may be associated with factors such as distribution of fat mass [74] and inflammatory status [75], the question remains are there simple life-style interventions than can improve metabolic health in the majority of the obese population? The current study suggests that, in the short term, this is not achieved by moderate increases fibre intake, either in RS or NSP form, above that currently recommended. There may be effects of such dietary interventions but these appear small compared with the improvements achieved by only a very modest weight loss. Of course, there may be additional beneficial effects on gut health, especially in the colon [15,72,76]. The careful nutritional control exerted in the current study was of only limited duration and other benefits may emerge from longer studies, as observed with low GI diets [12]. Furthermore, dietary fibre may alleviate the potential reduction in IS induced by certain nutrients, including protein [50]. Nonetheless, longer term weight loss also produces substantial benefits in terms of metabolic health [61] and so the route of reduced body mass should still be the main target of lifestyle intervention. 
